# Megahertz-rate ultrafast X-ray scattering and holographic imaging at the European XFEL

**DOI:** 10.1107/S1600577522008414

**Published:** 2022-09-29

**Authors:** Nanna Zhou Hagström, Michael Schneider, Nico Kerber, Alexander Yaroslavtsev, Erick Burgos Parra, Marijan Beg, Martin Lang, Christian M. Günther, Boris Seng, Fabian Kammerbauer, Horia Popescu, Matteo Pancaldi, Kumar Neeraj, Debanjan Polley, Rahul Jangid, Stjepan B. Hrkac, Sheena K. K. Patel, Sergei Ovcharenko, Diego Turenne, Dmitriy Ksenzov, Christine Boeglin, Marina Baidakova, Clemens von Korff Schmising, Martin Borchert, Boris Vodungbo, Kai Chen, Chen Luo, Florin Radu, Leonard Müller, Miriam Martínez Flórez, André Philippi-Kobs, Matthias Riepp, Wojciech Roseker, Gerhard Grübel, Robert Carley, Justine Schlappa, Benjamin E. Van Kuiken, Rafael Gort, Laurent Mercadier, Naman Agarwal, Loïc Le Guyader, Giuseppe Mercurio, Martin Teichmann, Jan Torben Delitz, Alexander Reich, Carsten Broers, David Hickin, Carsten Deiter, James Moore, Dimitrios Rompotis, Jinxiong Wang, Daniel Kane, Sandhya Venkatesan, Joachim Meier, Florent Pallas, Tomasz Jezynski, Maximilian Lederer, Djelloul Boukhelef, Janusz Szuba, Krzysztof Wrona, Steffen Hauf, Jun Zhu, Martin Bergemann, Ebad Kamil, Thomas Kluyver, Robert Rosca, Michał Spirzewski, Markus Kuster, Monica Turcato, David Lomidze, Andrey Samartsev, Jan Engelke, Matteo Porro, Stefano Maffessanti, Karsten Hansen, Florian Erdinger, Peter Fischer, Carlo Fiorini, Andrea Castoldi, Massimo Manghisoni, Cornelia Beatrix Wunderer, Eric E. Fullerton, Oleg G. Shpyrko, Christian Gutt, Cecilia Sanchez-Hanke, Hermann A. Dürr, Ezio Iacocca, Hans T. Nembach, Mark W. Keller, Justin M. Shaw, Thomas J. Silva, Roopali Kukreja, Hans Fangohr, Stefan Eisebitt, Mathias Kläui, Nicolas Jaouen, Andreas Scherz, Stefano Bonetti, Emmanuelle Jal

**Affiliations:** a Department of Physics, Stockholm University, 106 91 Stockholm, Sweden; b Max Born Institute for Nonlinear Optics and Short Pulse Spectroscopy, 12489 Berlin, Germany; c Institute of Physics, Johannes Gutenberg-University Mainz, 55099 Mainz, Germany; d European XFEL, Holzkoppel 4, 22869 Schenefeld, Germany; e Department of Physics and Astronomy, Uppsala University, Uppsala, Sweden; f Synchrotron SOLEIL, Saint-Aubin, Boite Postale 48, 91192 Gif-sur-Yvette Cedex, France; g Unité Mixte de Physique, CNRS, Thales, Université Paris-Saclay, 91767 Palaiseau, France; h Department of Earth Science and Engineering, Imperial College London, London SW7 2AZ, United Kingdom; i Faculty of Engineering and Physical Sciences, University of Southampton, Southampton SO17 1BJ, United Kingdom; j Max Planck Institute for the Structure and Dynamics of Matter, Luruper Chaussee 149, 22761 Hamburg, Germany; k Technische Universität Berlin, Zentraleinrichtung Elektronenmikroskopie (ZELMI), Berlin, Germany; l Institut Jean Lamour, Nancy, France; m Department of Materials Science and Engineering, University of California Davis, CA, USA; n Department of Physics, University of California San Diego, La Jolla, CA 92093, USA; o Center for Memory and Recording Research, University of California San Diego, La Jolla, CA 92093, USA; p MIREA – Russian Technological University, Moscow 119454, Russia; q Naturwissenschaftlich-Technische Fakultät – Department Physik, Universität Siegen, Siegen, Germany; r University of Strasbourg – CNRS, IPCMS, UMR 7504, 67000 Strasbourg, France; s Ioffe Institute, 26 Politekhnicheskaya, St Petersburg 194021, Russian Federation; t Sorbonne Université, CNRS, Laboratoire de Chimie Physique – Matière et Rayonnement, LCPMR, 75005 Paris, France; u Helmholtz-Zentrum Berlin für Materialien und Energie, 12489 Berlin, Germany; v Deutsches Elektronen-Synchrotron DESY, 22607 Hamburg, Germany; w Universität Hamburg, Hamburg, Germany; x Department of Physics and Astronomy, Aarhus University, Ny Munkegade 120, 8000C Aarhus, Denmark; y National Centre for Nuclear Research (NCBJ), A. Solłana 7, 05-400 Otwock-Świerk, Poland; z Department of Molecular Sciences and Nanosystems, Ca’ Foscari University of Venice, 30172 Venezia, Italy; aa Institute of Computer Engineering, Heidelberg University, Germany; bb Politecnico di Milano, Dipartimento di Elettronica, Informazione e Bioingegneria, 20133 Milano, Italy; cc Istituto Nazionale di Fisica Nucleare, Sezione di Milano, Milano, Italy; dd Dipartimento di Ingegneria e Scienze Applicate, Università degli Studi di Bergamo, Dalmine, Italy; ee Center for Free-Electron Laser Science CFEL, Deutsches Elektronen-Synchrotron DESY, Notkestraße 85, 22607 Hamburg, Germany; ff Diamond Light Source Ltd, Didcot, Oxfordshire OX11 0DE, United Kingdom; gg Center for Magnetism and Magnetic Materials, University of Colorado Colorado Springs, Colorado Springs, CO 80918, USA; hh Quantum Electromagnetics Division, National Institute of Standards and Technology, Boulder, CO, USA; ii Department of Physics, University of Colorado, Boulder, CO 80309, USA; jj Technische Universität Berlin, Institut für Optik und Atomare Physik, Berlin, Germany; kk Associate, Physical Measurement Laboratory, National Institute of Standards and Technology, Boulder, CO 80305, USA; University of Tokyo, Japan

**Keywords:** XFEL, holography, magnetic X-ray scattering, soft X-rays, ultrafast X-ray imaging

## Abstract

The advent of X-ray free-electron lasers (XFELs) has revolutionized fundamental science, from atomic to condensed matter physics, from chemistry to biology, giving researchers access to X-rays with unprecedented brightness, coherence and pulse duration. All XFEL facilities built until recently provided X-ray pulses at a relatively low repetition rate, with limited data statistics. Here, results from the first megahertz-repetition-rate X-ray scattering experiments at the Spectroscopy and Coherent Scattering (SCS) instrument of the European XFEL are presented. The experimental capabilities that the SCS instrument offers, resulting from the operation at megahertz repetition rates and the availability of the novel DSSC 2D imaging detector, are illustrated. Time-resolved magnetic X-ray scattering and holographic imaging experiments in solid state samples were chosen as representative, providing an ideal test-bed for operation at megahertz rates. Our results are relevant and applicable to any other non-destructive XFEL experiments in the soft X-ray range.

## Abstract 2

Results from the first megahertz-repetition-rate X-ray scattering experiments at the Spectroscopy and Coherent Scattering (SCS) instrument of the European XFEL are presented.

## 1. Introduction

X-rays have long been used as an advanced characterization tool of matter. They are typically used for diffraction, spectroscopy and imaging experiments with high spatial and energy resolutions. These properties have now been exploited for more than a century to achieve a deep understanding of molecules, solid materials and biological samples, fundamental to the progress of science. The appearance, one decade ago, of X-ray free-electron lasers (XFELs) providing intense X-ray pulses with a high degree of transverse spatial coherence and ultrashort pulses, has opened great opportunities for imaging and time-resolved experiments in atomic physics, condensed matter, chemistry and life sciences beyond what is possible at synchrotron light sources (Ayvazyan *et al.*, 2006 ▸; Emma *et al.*, 2010 ▸; Ishikawa *et al.*, 2012 ▸; Altarelli, 2011 ▸; Bostedt *et al.*, 2016 ▸; Grünbein *et al.*, 2018 ▸; Allaria *et al.*, 2012 ▸; Patterson *et al.*, 2010 ▸; Kang *et al.*, 2017 ▸; Halavanau *et al.*, 2019 ▸; Pellegrini, 2016 ▸).

XFEL technology constantly advances, particularly in terms of spectral brightness. The European XFEL (EuXFEL) is the first facility able to deliver soft and hard X-ray pulses at megahertz (MHz) repetition rate generated via a self-amplified spontaneous emission (SASE) process (Decking *et al.*, 2020 ▸). This greatly improves the statistics of the collected data and in turn the achievable signal-to-noise ratio within a typical experiment time. While in serial femtosecond X-ray crystallography many copies of the samples can be injected into the beam at MHz repetition rates for accumulation of data (Chapman *et al.*, 2011 ▸), it remains a challenge to recover or to replenish the sample for condensed matter studies in fields such as magnetism, strongly correlated materials and quantum science.

In this work, we demonstrate non-destructive, stroboscopic soft X-ray scattering and holography experiments at MHz repetition rates at the Spectroscopy and Coherent Scattering (SCS) beamline of the EuXFEL, exploiting the opportunities offered by the newly commissioned, custom-made two-dimensional detector able to match the EuXFEL MHz operation. We illustrate the initial capabilities of the beamline at the time of the presented experiments with representative examples of magnetic scattering and imaging experiments of the type performed at other XFELs (Vodungbo *et al.*, 2012 ▸; Pfau *et al.*, 2012 ▸; Graves *et al.*, 2013 ▸; Henighan *et al.*, 2016 ▸; Büttner *et al.*, 2017 ▸; Reid *et al.*, 2018 ▸; Dornes *et al.*, 2019 ▸; Malvestuto *et al.*, 2018 ▸; Weder *et al.*, 2020 ▸; Büttner *et al.*, 2021 ▸). We also estimate the heat load on the sample in these experiments, providing a figure-of-merit to find the optimal experimental parameters.

## 2. Results

### 2.1. Operation of the MHz-rate beamline and detector

**Figure 1:**
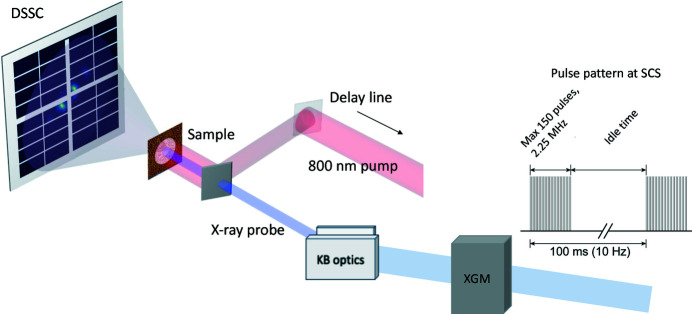
Schematic of the SCS beamline and of the X-ray pulse structure at the EuXFEL. The X-ray beam propagates from the right to the left side. The X-ray bursts arrive in trains which contain a user-defined number of pulses. The X-ray gas monitor (XGM) measures the pulse intensity *I*_0_ before the focusing KB optics. The pump laser is delivered into the experimental chamber via an auxiliary window and directed to the sample almost parallel with respect to the X-ray beam. The photons scattered by the sample are recorded on the DSSC detector.

At the EuXFEL, X-rays arrive in 10 Hz trains of multiple pulses. At the time of the experiment, the number of pulses within a train could be arbitrarily chosen between 1 and 150 separated by at least 440 ns, *i.e.* at a maximum repetition rate of 2.25 MHz within the train, see Fig. 1 ▸. The SCS beamline covers an energy range of 0.25 keV to 3 keV, well suited for core-level spectroscopy at the *L*-edges of 3*d* transition metals (including the most common ferromagnets), the *M*-edges of rare earth elements, the *K*-edges of lighter elements such as carbon, oxygen and sulfur, as well as the *L*-edges of some 4*d* metals. The photon energy can be changed via the undulator gap and synchronized with the soft X-ray monochromator. In addition, pulse energy tuning may be required for photon energy steps larger than 100 eV. A soft X-ray monochromator provides an energy resolution of approximately 250 eV for the Co and Fe absorption *L*-edges reported in this work (*E*/Δ*E* ≃ 3000), and reduces the pulse energy to tens of microjoules (Gerasimova *et al.*, 2022 ▸). The pulse duration of the monochromatic X-ray beam is 30 fs on average. See also Table I of the supporting information for key features of the SCS instrument..

As shown in Fig. 1 ▸, the incoming intensity *I*_0_ of each pulse is monitored by an X-ray gas monitor (XGM) (Maltezopoulos *et al.*, 2019 ▸). The beam size at the sample position can be adjusted using adaptive Kirkpatrick–Baez (KB) mirrors, providing a minimal spot diameter of approximately 1 µm and a maximum spot size of up to 500 µm in both horizontal and vertical directions (Mercurio *et al.*, 2022 ▸). Samples are mounted in the forward-scattering fixed target (FFT) chamber, which also includes an electromagnet that can be used to apply magnetic fields of up to 350 mT parallel to the X-ray beam direction. The SCS instrument is equipped with the novel DSSC (DEPFET Sensor with Signal Compression) detector, which can be positioned on a translation stage at given sample–detector distances over a range of 0.35 m to 5.40 m prior to the experiment. This allows users to cover different scattering wavevector ranges. During experiments the distance can be changed by 1.50 m around the working point. A multichannel-plate-based transmission intensity monitor (not shown in Fig. 1 ▸) simultaneously collects the direct beam after the DSSC detector and is used to measure the sample absorption. The pump laser beam is inserted in the FFT experiment station with an in-coupling mirror and impinges on the sample nearly collinearly with the X-rays. The laser used here is a YAG-white-light-seeded, non-collinear optical parametric amplifier developed in-house at the EuXFEL providing pump pulses of 800 nm with a duration down to 35 fs, which can match the pulse pattern of the XFEL (Pergament *et al.*, 2016 ▸; Palmer *et al.*, 2019 ▸). The incoming pulse energy can be adjusted from 0.05 mJ up to 2 mJ per pulse with a spot size from tens to hundreds of micrometres in diameter. Spatial overlap between the optical and X-ray beams is achieved by monitoring the beam position on boron nitride in the plane of the sample. Temporal overlap is achieved by looking at the optical reflectivity change of an Si_3_N_4_ sample upon X-ray excitation. The delay between the optical pump and X-ray probe can be changed by up to 1 ns using a mechanical delay line. Larger delays can be selected using a phase shifter or the trigger system. In this work, the sample is always pumped at half the probe repetition frequency in order to obtain pairs of pumped and unpumped measurements that are close in time. This allows users to remove the effect of long-term drift on the measurements.

The DSSC is presently the fastest 1 Mpixel camera available worldwide, providing single-photon sensitivity in the soft X-ray regime. The present camera uses for each hexagonal pixel a miniaturized silicon drift detector (MiniSDD) coupled to a linear readout electronics front-end, while a second version will employ non-linear DEPFET active pixel sensors (Porro *et al.*, 2021 ▸). The DSSC detector is capable of recording data from the full pixel array with a 220 ns frame interval, corresponding to a 4.5 MHz repetition rate. The data are retrieved in the 10 ms-long inter-pulse train gap of the XFEL. The sensitive area of the camera is about 505 cm^2^ in size, composed of 1024 × 1024 equilateral hexagonal pixels with a side length of 136 µm. The camera comprises 16 sub-units called ‘ladders’ (horizontal blocks) arranged into four quadrants. Each ladder has two monolithic sensors and is read out by 16 independent readout application-specific integrated circuits (ASICs) (Porro *et al.*, 2021 ▸). The four quadrants can be moved independently if required by the experiment, while the location of the ladders within one quadrant is fixed.

**Figure 2:**
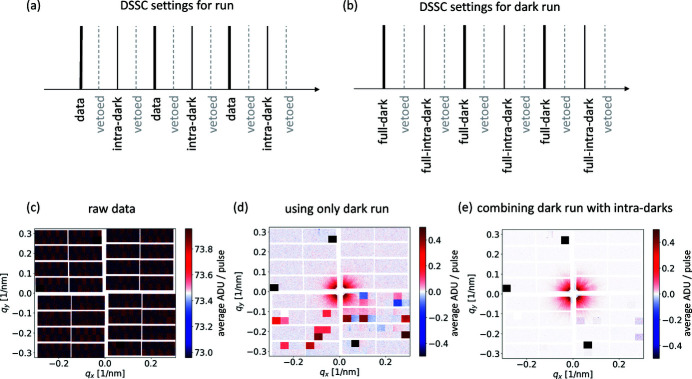
Schematic of the pulse labeling for the dark subtraction and application example. X-ray pulse labeling for acquisition (*a*) with X-rays and (*b*) without X-rays, a so-called *dark run*. Separate dark runs are usually 1 min for practical reasons (here 90000 frames). (*c*) Raw data collected by the DSSC detector plotted around its mean value. (*d*) Dark subtraction using only a separate dark run and (*e*) dark subtraction combining a separate dark run and the intra-dark events.

While the DSSC detector always runs at 4.5 MHz, a ‘veto’ system allows frames to be discarded according to a user-defined pattern or an additional signal provided by an external veto source. When pulses are delivered at a smaller frequency than 4.5 MHz, the user can choose to record frames at the same frequency as the XFEL. Discarding (vetoing) unused frames is crucial to minimize the amount of data collected and perform efficient analysis. In fact, at full repetition rate, the camera produces a data rate of 134 Gbit s^−1^, which leads to single experiments creating petabytes of data. So-called *intra-dark* frames, see Figs. 2 ▸(*a*)–2(*b*), are regularly collected in between data frames to improve the contrast in the final image as described in the next paragraph. Fig. 2 ▸(*c*) is an example of the raw data collected by the DSSC detector; the uncorrected image has a mean of 73.35 ADUs, which is almost entirely an offset signal due to the analog-to-digital converters (Hansen *et al.*, 2013 ▸; Porro *et al.*, 2021 ▸), which can be removed by appropriate signal subtraction.

The first dark signal subtraction, pixel-by-pixel, is made using dark frames acquired in a separate run with the same settings of the DSSC camera (gain and veto pattern), but without X-rays hitting the detector. This is labeled as a *dark run*, and subtraction of such a run from the data results in the plot in Fig. 2 ▸(*d*). The few darker squares in the figure are due to the fact that, for a few random frames, the ASICs did not transfer the acquired data correctly. This is due to a firmware bug that was solved after the experiment. A separate dark run helps to remove the large static electronic offset, but does not correct for other sources of noise, such as the signal-generated backscattered photons or other systematic electronic effects which are occurring during the measurements. These can, however, be removed using the intra-dark signal, closer in time to the signal events. By combining the dark run with the intra-darks, one can achieve the most appropriate background subtraction, as shown in Fig. 2 ▸(*e*), where the image was calculated as

[run(data frame − intra-dark frame)] − [dark run(data frame − intra-dark frame)].

Note that the three black squares indicate ASICs that were damaged and cannot be used for data collection (Porro *et al.*, 2021 ▸). We estimate an experimental root-mean-square (RMS) noise for each pixel: σ/*N* ^1/2^ ≃ 5 × 10^−3^ ADU, where σ ≃ 1.4 ADU is the standard deviation and *N* ≃ 10^5^ is the number of events in a measurement run. With the four data sets needed for complete offset subtraction, this leads to a total RMS noise of σ_tot_/*N* ^1/2^ ≃ 10^−2^ ADU, which allows to readily measure signals in the 0.1–1 ADU range, as shown in Fig. 2 ▸(*e*).

During the beam time, more than 780 terabytes of data were captured using the EuXFEL’s control and acquisition system (Hauf *et al.*, 2019 ▸). Offline data analysis was directed from Python and Jupyter notebooks (Fangohr *et al.*, 2020 ▸), making use of the storage, calibration, compute and data analysis infrastructure at EuXFEL (Kuster *et al.*, 2014 ▸; Fangohr *et al.*, 2018 ▸). Analysis tools that were developed for this work and that can be re-used for similar research have been integrated into the EuXFEL open-source software data analysis stack (Fangohr *et al.*, 2022 ▸).

### 2.2. Ultrafast small-angle X-ray scattering at MHz repetition rates

Small-angle X-ray scattering (SAXS) in the soft X-ray regime has been shown to be a unique tool to explore not only the temporal but also the spatial dynamics of ultrafast processes on nanometre length scales. In ultrafast magnetism, this capability has been proven to be a crucial feature, since many of the fundamental physical processes at play are strongly connected to the nanometre structure in the material (Vodungbo *et al.*, 2012 ▸; Pfau *et al.*, 2012 ▸; Graves *et al.*, 2013 ▸; Bergeard *et al.*, 2015 ▸; Iacocca *et al.*, 2019 ▸; Hennes *et al.*, 2020 ▸). We measure thin film multilayers with a composition of Ta(3 nm) / Cu(5 nm) / [CoFe(0.25 nm)–Ni(0.75 nm)]_20_ / CoFe(0.25 nm) / Cu(3 nm) / Ta(3 nm) deposited on 200 nm-thick Si membranes with a lateral size of 2 mm. Sample thicknesses were calibrated with X-ray reflectometry. This CoFe/Ni multilayer sample has an out-of-plane magnetization showing ordered stripe domains with a typical domain size in the range 115–125 nm, as revealed by magnetic force microscopy (see the supporting information). The magnetic domains were aligned to stripes after in-plane demagnetization and were characterized via SAXS at the VEKMAG endstation at the BESSY II synchrotron (Noll & Radu, 2017 ▸) and at the RESOXS endstation of the SEXTANTS beamline at Synchrotron SOLEIL, as well as by magnetic force microscopy.

Due to the X-ray magnetic circular dichroism (XMCD) effect, the magnetic stripe domains act as an absorption grating for linearly polarized photons in resonance with the Co *L*_3_ absorption resonance at approximately 778 eV (Hellwig *et al.*, 2003 ▸). This gives rise to an anisotropic scattering signal along a preferential axis. The sample also comprises a curved diffraction grating milled in the silicon carrier membrane, using a focused Ga^+^ ion beam (FIB) system, creating a non-resonant reference scattering signal on the detector (Schneider *et al.*, 2016 ▸). The DSSC camera is placed 2 m from the sample and the X-ray beam size is 75 µm. As optical pump, we use 800 nm, 100 fs laser pulses with a spot size of 370 µm. The pump laser is operated at a repetition rate of 282 kHz with 10 pulses per train, while the XFEL runs at 564 kHz with 20 pulses per train, allowing unpumped X-ray scattering frames to be recorded in between pumped ones. Due to thermal damage, the number of optical laser pulses had to be limited to 10 pulses per train, which led to a total of 20 X-ray pulses per train to record pumped and unpumped data. For static measurements, the number of X-ray pulses could be increased to 50 per train.

**Figure 3:**
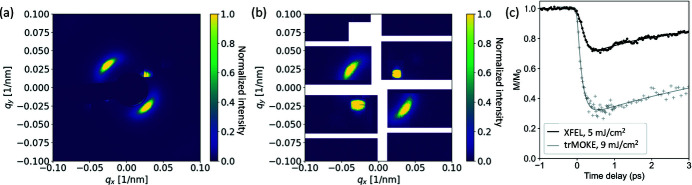
MHz-rate time-resolved magnetic X-ray scattering. Resonant Co *L*_3_-edge scattering pattern of a CoFe/Ni thin film multilayer recorded at (*a*) the SEXTANTS beamline at Synchrotron SOLEIL and (*b*) the SCS beamline at the EuXFEL. The first-order magnetic scattering is observed along the top-left to bottom-right diagonal. The scattering from the non-magnetic grating is the feature visible along the opposite diagonal. The intensity is in linear scale and normalized to the maximum magnetic scattering amplitude. (*c*) Time-resolved pump–probe data recorded on the same sample. Black symbols: data from the EuXFEL, computed as the azimuthally integrated intensity of the first-order peak in the frames when the pump laser was impinging on the sample, divided by the nearest previous unpumped frame. Gray symbols: data from a table-top MOKE setup with different pump fluence. The solid lines show the fit to the data. Further details are given in the main text.

A typical scattering pattern from the magnetic stripe domains recorded from the SEXTANTS beamline at Synchrotron SOLEIL (Sacchi *et al.*, 2013 ▸) is shown in Fig. 3 ▸(*a*), with the corresponding XFEL data in Fig. 3 ▸(*b*). In both images, we observe two broad features arising from the scattering of X-rays from the magnetic domains along the top-left/bottom-right diagonal of the image, as well as the smaller features related to the reference diffraction grating along the opposite diagonal. The synchrotron image is acquired with an average photon rate of 5 × 10^12^ photons s^−1^ and 1 s exposure time while for the XFEL data a total of 9 × 10^11^ photons were incident on the sample, with 50 pulses per train and 600 trains in total with an average of 3 × 10^7^ photons pulse^−1^. Note that the repetition rate of SOLEIL is 325 MHz, which gives roughly 1.6 × 10^4^ photons pulse^−1^.

The black symbols in Fig. 3 ▸(*c*) show the laser-induced ultrafast dynamics of the magnetic scattering spot intensities, measured in a pump–probe configuration, with a pump fluence of 5 mJ cm^−2^ and with the sample at magnetic remanence. In the same plot, we compare the XFEL data with those recorded on the very same sample using a table-top time-resolved magneto-optical Kerr effect (tr-MOKE) setup with a saturating magnetic field and with a pump fluence of 9 mJ cm^−2^. Both curves describe the laser-induced ultrafast demagnetization of the ferromagnetic film (Beaurepaire *et al.*, 1996 ▸). The curves were fitted using the formula derived from a three-temperature model (Beaurepaire *et al.*, 1996 ▸; Malinowski *et al.*, 2008 ▸; Hennes *et al.*, 2020 ▸), *i.e.*


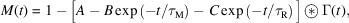

where τ_M_ is the demagnetization time and τ_R_ is the picosecond recovery time, different from the thermal one with much larger time constant. The constants *A*, *B* and *C* are amplitudes that can be related to the different physical processes. Here we are only interested in the time constants, and we neglect further considerations on these amplitudes. The convolution with a Gaussian function Γ(*t*) takes into consideration the finite pulse durations which were different for the tr-SAXS and tr-MOKE measurements, and allows us to extract the true demagnetization constant. From the fit of the XFEL data, we find τ_M_ = 102 ± 8 fs and τ_R_ = 2.18 ± 0.07 ps, while from the tr-MOKE we obtain τ_M_ = 129 ± 10 fs and τ_R_ = 6.08 ± 0.5 ps. The slightly smaller time constants retrieved for the XFEL measurements are consistent with a smaller quenching of the sample (Koopmans *et al.*, 2010 ▸). Note that the good signal-to-noise ratio of the XFEL data indicates that normalization of the DSSC data with the XGM signal is reliable (see the supporting information for details), opening the way to high-quality spectroscopy experiments not achievable at earlier XFELs (Higley *et al.*, 2016 ▸; Tiedtke *et al.*, 2014 ▸).

### 2.3. X-ray holographic imaging at MHz repetition rates

High-resolution X-ray imaging techniques are mostly of two kinds: those based on Fresnel-type optics, and those which are lensless. While the former type has found much application at synchrotron lightsources, they are difficult to realize in the soft X-ray region at free-electron lasers due to the risk of damage by strong absorption of intense X-ray pulses. In these facilities, lensless techniques are preferred for full field imaging, since they can exploit the high degree of transverse coherence of XFEL radiation (Wang *et al.*, 2012 ▸; von Korff Schmizing *et al.*, 2014 ▸; Willems *et al.*, 2017 ▸). X-ray holography is one such lensless imaging technique that relies on the interference between two beams, where one holds information about the sample, and the other acts as the phase reference. A Fourier transform of the two-dimensional diffraction reconstructs the real-space image. The samples are magnetic multilayer films of [Ta(5 nm)/Co_20_Fe_60_B_20_(0.9 nm)/MgO(2 nm)]_15_ with out-of-plane magnetization. They were produced by DC magnetron sputtering deposition on Si_3_N_4_ membranes. From magnetic force microscopy we observe approximately 200 nm-wide labyrinth magnetic domains at remanence. The holography aperture is a square with a side of 2.5 µm, rotated by 45° with respect to the sides of the X-ray transparent window where the film is deposited. The reference beam is generated by two orthogonal slits in the holography mask (see the supporting information for details). This allows to reconstruct the image using the HERALDO technique (Zhu *et al.*, 2010 ▸; Duckworth *et al.*, 2011 ▸), which mitigates the artifacts due to the detector gaps. The HERALDO holography mask was fabricated by milling reference slits through the 1 µm-thick Au layer using an FIB system. These reference slits (40 nm wide and 4 µm long) are milled through the Au, the Si_3_N_4_ membrane and the magnetic thin film while only the Au is removed over the sample (object hole).

**Figure 4:**
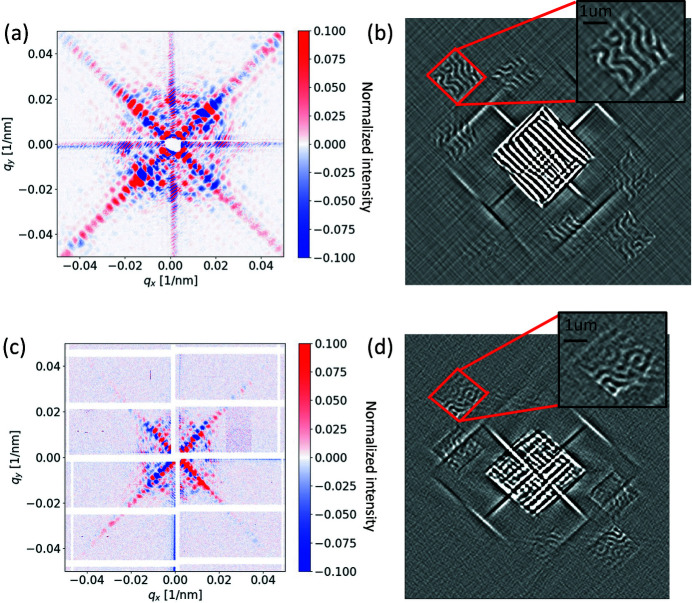
Megahertz-rate magnetic X-ray holographic imaging. Magnetic hologram of a CoFeB thin film multilayer recorded at the Fe *L*_3_-edge at (*a*) the COMET endstation at the SEXTANTS beamline at Synchrotron SOLEIL and (*c*) the SCS beamline at the EuXFEL at a 2.25 MHz repetition rate. The intensity is in linear scale and normalized to the maximum intensity value. Reconstructions of the magnetic domains using the HERALDO technique on (*b*) the synchrotron data and (*d*) the free-electron laser data.

The sample was pre-characterized at the COMET endstation at SOLEIL (Popescu *et al.*, 2019 ▸). In Fig. 4 ▸(*a*) we plot the magnetic scattering signal recorded at the synchrotron, calculated as the difference between the signal taken with X-rays of opposite helicities at the Fe *L*_3_-edge, *i.e.* at approximately 707 eV. In Fig. 4 ▸(*b*), we show the corresponding image reconstruction applying the full HERALDO procedure (Zhu *et al.*, 2010 ▸; Duckworth *et al.*, 2011 ▸). The image reveals the presence of magnetic domains in one of the six smaller squares which are the cross-correlation between the object and the three corners of the L-shaped reference slit. Each corner yields a pair of conjugated images, where the opposite contrast indicates oppositely oriented magnetic domains. The XFEL measurement on the same sample – with different magnetic domain pattern due to exposure to a magnetic field between the respective measurements – is shown in Fig. 4 ▸(*c*), where in this case the X-ray helical polarization at the required photon energy is achieved with a thin Fe film polarizer inserted into the beam before the sample (Müller *et al.*, 2018 ▸), at the expense of photon flux. Helicity reversal is obtained by reversing the magnetic field applied to the thin film polarizer. The detector is placed 4.6 m away from the sample, in order to record the magnetic information in the lower *q*-range. The beam spot is 50 µm in diameter, smaller than in the case of the SAXS experiment, but much larger than the holography apertures. The samples are probed with different repetition rates of the XFEL between 0.226 MHz and 2.25 MHz with no sample damage observed. This can be partly explained by the thick gold layer where the holography mask is patterned, as we discuss in detail in the final part of this work. The hologram is the result of 15 min acquisition (1000 pulses s^−1^) corresponding to 4 × 10^13^ photons on the sample area for each helicity. As a comparison, the photon count on the same sample area at the COMET endstation of the SEXTANTS beamline was 1 × 10^13^ photons acquired in 90 s. Fig. 4 ▸(*d*) shows the 2D Fourier transform of the hologram of the XFEL data. Like in Fig. 4 ▸(*b*) we observe the auto-correlation of the object aperture in the center of the image, and three pairs of reconstructions.

## 3. Discussion

When comparing the SAXS measurements in Fig. 3 ▸, we note that the number of pulses per train had to be reduced to 50 in order to keep the sample unchanged by the X-rays; subsequently the average photon flux (photons s^−1^) is two orders of magnitude smaller compared with that of the synchrotron, mostly limited by the burst mode operation of the machine. Naturally, the XFEL measurements are performed using femtosecond X-ray pulses, which allows for ultrafast experiments that are not feasible at a synchrotron. We have also confirmed that the extracted time constants with table-top and XFEL experiments are comparable, demonstrating the reliability of the XFEL measurements in measuring ultrafast dynamics.

Looking at the holographic imaging data we notice that, despite the fact that the XFEL image is slightly noisier, the magnetic domains are clearly distinguishable. We believe that part of the issue is also a non-ideal illumination of the holographic mask which can be readily improved with an optimized design. Furthermore, the slight distortion in the reconstruction is due to a simplified hexagonal-to-Cartesian pixels conversion that does not include sub-pixel interpolation. Nevertheless, these data demonstrate that a full magnetic image reconstruction at the EuXFEL is possible within tens of minutes. Hence, ‘movies’ of the magnetization on ultrafast time-scales and nanometre resolutions are now possible at an XFEL within a typical beam time allocation. The availability of an afterburner generating circularly polarized X-rays will increase the polarization degree from 50% (thin film polarizers) to 100% and enhance the signal-to-noise of the charge–magnetic interference term which is responsible for magnetic contrast in the image reconstructions. Altogether, these improvements potentially shorten the acquisition time by up to one order of magnitude.

**Figure 5:**
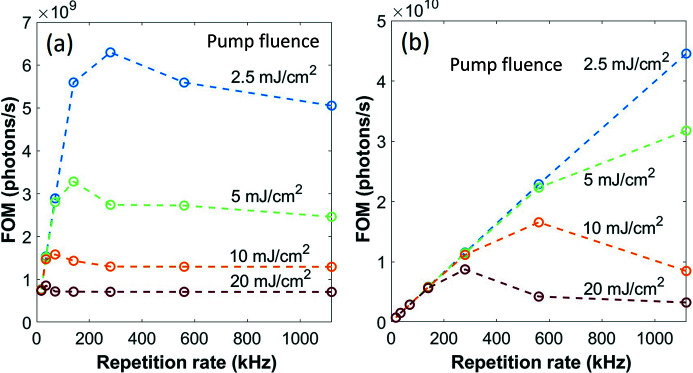
Figure of merit for magnetic scattering as a function of repetition rate of XFEL pulses. Simulated photons per second of magnetic scattering as a function of XFEL repetition rate in a burst of 280 µs and for different pump fluences: (*a*) bare thin-film samples on Si membranes and (*b*) samples on Si membranes with an additional 500 nm heat sink layer. Note that the FOM scales are different in (*a*) and (*b*) by a factor of ten.

Finally, we estimate the possible heating effects of X-ray pulses at high repetition rate on the samples. We perform heat diffusion simulations (Appendix *A* [App appa]), and we use the dependence of the magnetization on temperature to calculate the loss of signal due to heat. The details are given in Appendix *A* [App appa]. These calculations allow us to obtain a figure of merit (FOM) which can then be plotted as a function of XFEL repetition rate (considering the actual pulse structure), and for different pump fluences, as shown in Figs. 5 ▸(*a*)–5(*b*). The FOM is determined by the competition of two processes: the number of photons reaching the detector, which increases linearly with the average X-ray power, and the amount of meaningful signal (proportional to the magnetization squared), which decreases with average power. Thus, the FOM can be interpreted as the number of information-carrying photons hitting the detector over a given time. We find that the optimal repetition rate is of the order of 100 kHz for pump–probe measurements on typical samples on free-standing membranes, which can be pushed to the MHz rate if a proper heat sink layer is implemented within the sample, such as for the case of holographic imaging experiments.

## 4. Related literature

The following references, not cited in the main body of the paper, have been cited in the supporting information: Avery *et al.* (2015 ▸); Chen & Hui (1999 ▸); Costescu *et al.* (2003 ▸); Gundrum *et al.* (2005 ▸); Henke *et al.* (1993 ▸); Johnson & Christy (1974 ▸); Kimling *et al.* (2017 ▸); McConnell *et al.* (2001 ▸); McPeak *et al.* (2015 ▸); Ordal *et al.* (1985 ▸, 1987 ▸, 1988 ▸); Saldin *et al.* (2010 ▸); Schinke *et al.* (2015 ▸); Srichandan (2018 ▸); Zhu *et al.* (2012 ▸).

## Supplementary Material

Table summarizing the SCS instrument features, work on the DSSC-XGM intensity correlation, details about the X-ray holography sample and the Heraldo technique, and details of the heat diffusion simulation. DOI: 10.1107/S1600577522008414/ay5598sup1.pdf

## Appendix A Heat diffusion simulation

The fraction of X-ray and optical pulse energy absorbed by the CoFe/Ni multilayered sample was calculated using the optical constants and refractive indexes of the sample materials for certain photon energies, available from online databases. The subsequent heat diffusion in the layers was simulated with the equation

The first and second terms of equation (1) describe the heat diffusion in the layer *n*, while the third term introduces the heat exchange between the layers *n* and *n* + 1. In equation (1), *T* is the temperature of the layer *n*, ρ is the mass density, *C* is the heat capacity and *k* is the thermal conductivity of the respective layer, and *h* is the coefficient of heat transfer between layers *n* and *n* + 1. The *h* value depends on the thermal conductivity and the thickness of two layers, as well as the thermal conductance of the interface between them (Lyeo & Cahill, 2006 ▸). Equation (1) was solved numerically for each layer of the sample in the polar coordinates. We assumed that the system is two-dimensional since the thickness of the layers is much smaller than their lateral size. In the heat diffusion simulation, the lateral sample size was 2 mm, the spacing for the computation grid was 2 µm, the total time of the simulation 270 µs and the time step 1.35 ps. While varying the X-ray and pump pulses repetition rate, the pump was always kept at half the frequency of the X-ray probe. We used the constant temperature boundary conditions, assuming the perfect heat removal from the sample by the perimeter, which is always maintained at room temperature. All parameters of the simulation were taken as constants at room temperature. The magnetization was estimated from the temperature values using the mean field approximation (Kittel *et al.*, 1996 ▸),

where *T* and *M* are the average temperature and magnetization of the magnetic layers within the X-ray beam spot, *T*_C_ = 750 K is the Curie temperature of the CoFe/Ni sample and *M*_S_ = 1 is the saturation magnetization.
