# Supplementary material for: Megahertz-rate ultrafast X-ray scattering and holographic imaging at the European XFEL
Source: J Synchrotron Radiat. 2022 Sep 29;29(Pt 6):1454–64. doi: 10.1107/S1600577522008414 (PMC9641564; doi:10.1107/S1600577522008414)
Supplement: Supplementary file 1 [file s-29-01454-sup1.pdf]

JOURNAL OF SYNCHROTRON RADIATION

Volume 29 (2022)

Supporting information for article:

Megahertz-rate Ultrafast X-ray Scattering and Holographic Imaging at the European XFEL

Nanna Zhou Hagström, Michael Schneider, Nico Kerber, Alexander Yaroslavtsev, Erick Burgos Parra, Marijan Beg, Martin Lang, Christian M. Günther, Boris Seng, Fabian Kammerbauer, Horia Popescu, Matteo Pancaldi, Kumar Neeraj, Debanjan Polley, Rahul Jangid, Stjepan B. Hrkac, Sheena K. K. Patel, Sergei Ovcharenko, Diego Turenne, Dmitriy Ksenzov, Christine Boeglin, Marina Baidakova, Clemens von Korff Schmising, Martin Borchert, Boris Vodungbo, Kai Chen, Chen Luo, Florin Radu, Leonard Müller, Miriam Martínez Flórez, André Philippi-Kobs, Matthias Riepp, Gerhard Grübel, Robert Carley, Justine Schlappa, Benjamin E. Van Kuiken, Rafael Gort, Laurent Mercadier, Naman Agarwal, Loïc Le Guyader, Giuseppe Mercurio, Martin Teichmann, Jan Torben Delitz, Alexander Reich, Carsten Broers, David Hickin, Carsten Deiter, James Moore, Dimitrios Rompotis, Jinxiong Wang, Daniel Kane, Sandhya Venkatesan, Joachim Meier, Florent Pallas, Tomasz Jezynski, Maximilian Lederer, Djelloul Boukhelef, Janusz Szuba, Krzysztof Wrona, Steffen Hauf, Jun Zhu, Martin Bergemann, Ebad Kamil, Thomas Kluyver, Robert Rosca, Michał Spirzewski, Markus Kuster, Monica Turcato, David Lomidze, Andrey Samartsev, Jan Engelke, Matteo Porro, Stefano Maffessanti, Karsten Hansen, Florian Erdinger, Peter Fischer, Carlo Fiorini, Andrea Castoldi, Massimo Manghisoni, Cornelia Beatrix Wunderer, Eric E. Fullerton, Oleg G. Shpyrko, Christian Gutt, Cecilia Sanchez-Hanke, Hermann A. Dürr, Ezio Iacocca, Hans T. Nembach, Mark W. Keller, Justin M. Shaw, Thomas J. Silva, Roopali Kukreja, Hans Fangohr, Stefan Eisebitt, Mathias Kläui, Nicolas Jaouen, Andreas Scherz, Stefano Bonetti and Emmanuelle Jal

MHz-rate Ultrafast X-ray Scattering and Holographic Imaging at the European XFEL

–

Supplementary Information

I. SCS INSTRUMENT FEATURES

Photon energy	0.25 keV to 3 keV
Relative photon bandwidth	0.5%
X-ray pulse energy	≤10 mJ
X-ray pulse duration	1 fs to 100 fs (FWHM)
Polarization	Linear. Elliptical with thin film polarizers.
Focal spot size on the sample	From 1 µm (horizontal and horizontal) to 500 µm
Magnetic field	DC electromagnet ≤ 0.5T
Optical laser systems	800 nm: ≤1 mJ, 15 fs to 100 fs<br>1030 nm: ≤20 mJ, 850 fs

TABLE I. Key features of the SCS instrument available for the experiments described in the main text. Future developments are not discussed in the current manuscript.

II. DSSC–XGM INTENSITY CORRELATION

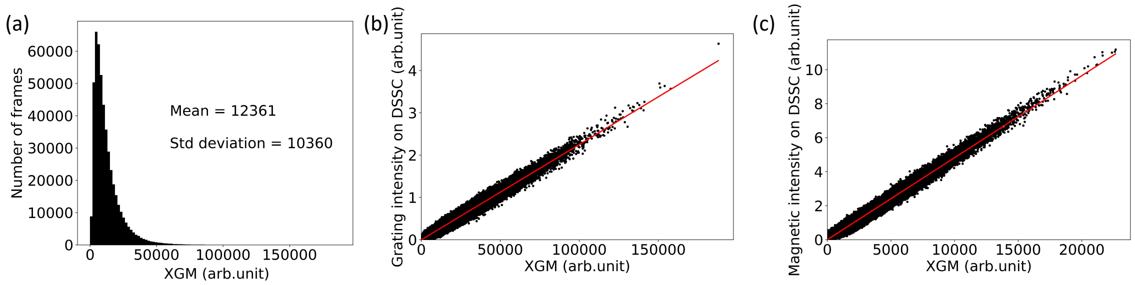

FIG. 1. **X-ray pulse intensity fluctuation and correlation with the DSSC detector data.** (a) Histogram of the single-shot X-ray intensities within one typical acquisition run containing $5 \cdot 10^5$ pulses measured with the XGM I_0 detector. (b) Symbols: charge scattering amplitude from a curved grating measured on the DSSC detector as a function of I_0. Solid line: linear regression on the data with a coefficient of determination $R^2 = 0.95$. (c) Symbols: magnetic scattering amplitude from magnetic domains measured on the DSSC detector as a function of I_0. Solid line: linear regression on the data with a coefficient of determination $R^2 = 0.97$.

The X-ray pulses produced at XFELs based on the SASE mechanism have intrinsic intensity fluctuations due to the stochastic nature of the SASE process [1]. For this reason, it is important to monitor the incoming pulse intensity I_0 on a shot-to-shot basis using an XGM, and properly normalize the 2D detector signal. A histogram of I_0 measured by the XGM for a typical XFEL run is shown in Fig. 1(a). One can clearly see that the standard deviation of the curve is comparable to its mean, which justifies the need for a shot-to-shot normalization. In addition, since two different detectors are used to measure the incoming and the scattered X-ray intensity, it must be ensured that both operate in a regime away from intrinsic nonlinearities, which are typically different for different detectors. In Fig. 1(b)-(c) we show the intensity correlation between the DSSC detector and the I_0 monitor for two types of samples. In Fig. 1(b), we used custom-built curved gratings [2], which created a pair of isolated scattering features on the DSSC. The y-axis is the integrated intensity over a suitable region of the detector which contains those isolated features. In Fig. 1(c) instead, we used a sample comprising labyrinth magnetic domains producing a ring-like scattering pattern on the DSSC detector. The y-axis in this case is the azimuthally integrated intensity over the entire ring. For both the curved grating and the magnetic domain sample, with one order of magnitude different I_0 values, the XGM and DSSC detectors show a clear linear correlation. This indicates that normalization of the DSSC data with the XGM signal is reliable, opening the way to high quality spectroscopy experiments not achievable at earlier XFELs.

III. TIME-RESOLVED SAXS SAMPLE DETAILS

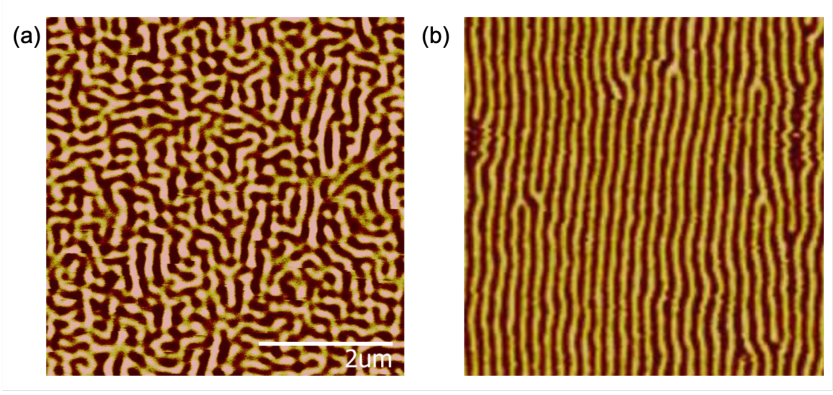

FIG. 2. (a) 5 µm x 5 µm MFM images of the CoFe/Ni multilayer. (b) Example of a MFM image of stripe domains aligned with an external in-plane magnetic field.

Figure 2(a) shows the MFM of the Ta(3 nm) / Cu(5 nm) / $[CoFe(0.25\,nm)\text{-}Ni(0.75\,nm)]_{20}$ / CoFe(0.25 nm) / Cu(3 nm) / Ta(3 nm) multilayer sample. This image shows randomly oriented labyrinth domains. Those were aligned into stripes with an in-plane magnetic field. A typical MFM image of the stripe domains obtained in this way is shown in Fig. 2(b).

IV. X-RAY HOLOGRAPHY SAMPLE DETAILS

Figure 3(a) illustrates a MFM image of the $[Ta(5\,nm)/Co_{20}Fe_{60}B_{20}(0.9\,nm)/MgO(2\,nm)]_{15}$ multilayer sample. Figure 3(b) shows a SEM image of the holography mask. The mask is a square with a 2.5 µm side, rotated by 45° with respect to membrane's side. The holography mask has also two orthogonal slits for encoding the reference beam, according to the HERALDO technique [3].

V. RECONSTRUCTION USING THE HERALDO TECHNIQUE

In this section we show the impact of the inactive areas of the DSSC on the holographic image reconstruction. Fig 4(a) shows the hologram recorded at the SOLEIL synchrotron mapped with the gaps of the DSSC and the corresponding reconstructed image is shown in Fig 4(b). While the gaps generally affect the reconstruction, this holography mask was specifically designed to minimize their impact, as clearly shown in the figure. A more crucial aspect for the reconstruction is the correct placement of the 16 independent ladders of the detector, since a small displacement of one single ladders can severely distort the reconstruction from Fourier

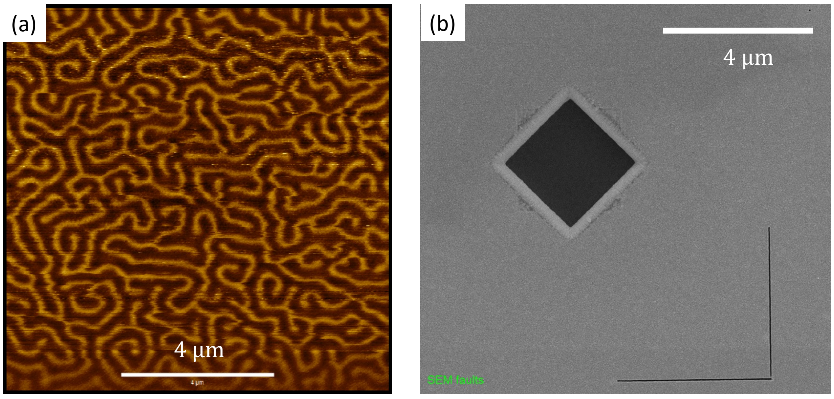

FIG. 3. (a) 10 µm x 10 µm MFM image of the CoFeB multilayer sample discussed in the main text. (b) Scanning Electron Microscope (SEM) image of the holography mask.

transform. A on-time systematic and careful procedure to manually align the quadrants using the characteristic features of the scattering was required ahead of the first reconstruction, but once the detector is aligned, it is ready for all subsequent data and experiments.

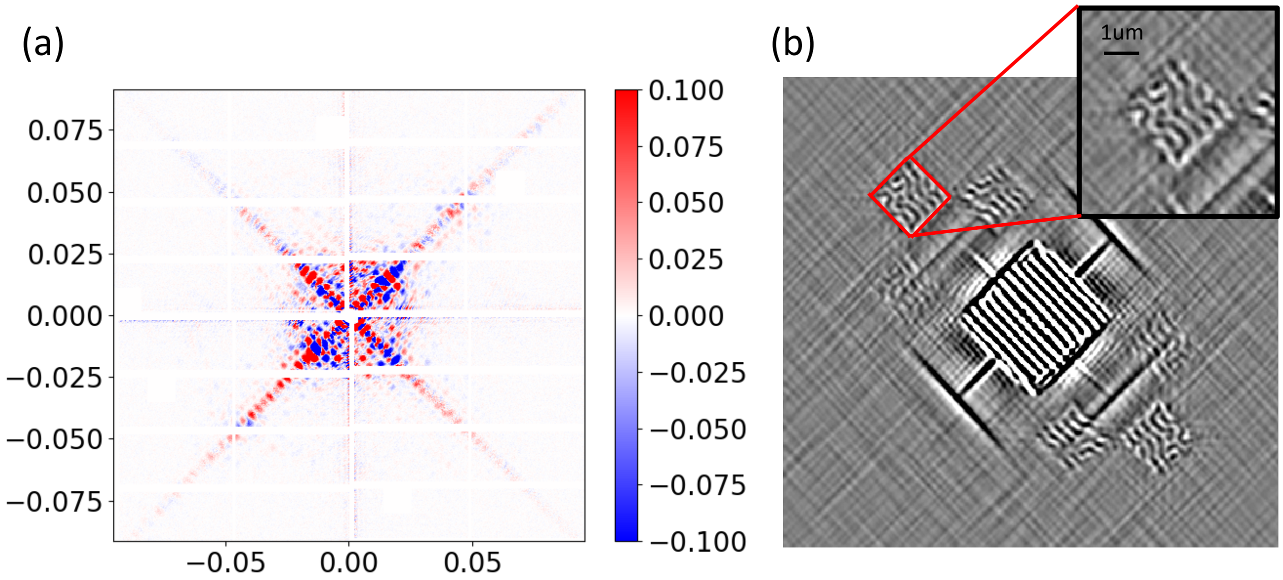

FIG. 4. (a) Hologram recorded at the COMET-end station at the SOLEIL synchrotron masked with the gaps of the DSSC detector. (b) Reconstructions of the magnetic domains using the HERALDO technique.

VI. HEAT DIFFUSION SIMULATION DETAILS

The parameters used for the heat diffusion simulation are summarized in Table II. In addition to those, the thermal conductance of the layer-to-layer interfaces are assumed to be $2 \times 10^9\,\mathrm{W\,m^{-2}\,K^{-1}}$ for the metal-metal and $0.3 \times 10^9\,\mathrm{W\,m^{-2}\,K^{-1}}$ for the metal-insulator cases [4–6].

	n	k	β [7]	Thermal conductivity $\mathrm{W\,m^{-1}\,K^{-1}}$	Heat capacity $\mathrm{J\,kg^{-1}\,K^{-1}}$	Density $\mathrm{kg\,m^{-3}}$
Ta	1.11 [8]	3.48 [8]	1.26×10^{-3}	57.5	140	16690
Cu	0.105 [9]	5.14 [9]	2.97×10^{-3}	41 [10]	385	8960
$[\mathrm{CoFe/Ni}]_{20}$	2.49 [11–13]	4.51 [11–13]	1.96×10^{-3}	42.8 [14, 15]	435	8877
Si	3.67 [16]	0.005 [16]	9.46×10^{-5}	10 [17]	691 [18]	2329
Au	0.18 [12]	5.15 [12]	1.95×10^{-3}	244.3 [19]	129	19300

TABLE II. Parameters used for the heat diffusion simulation. The refractive index n and extinction coefficient k is for 800 nm wavelength, while the X-ray extinction coefficient β is taken for the photon energy of the cobalt L_3 edge 778.8 eV. The parameters of the $[\mathrm{CoFe(0.25\,nm)\,/\,Ni(0.75\,nm)}]_{20}$ multilayer sample have been extrapolated using the parameters of the single constituting elements.

[1] E. L. Saldin, E. A. Schneidmiller, and M. V. Yurkov, New Journal of Physics **12**, 035010 (2010), ISSN 1367-2630, URL https://doi.org/10.1088/1367-2630/12/3/035010.

[2] M. Schneider, C. M. Günther, C. v. K. Schmising, B. Pfau, and S. Eisebitt, Optics Express **24**, 13091 (2016), ISSN 1094-4087, URL https://www.osapublishing.org/oe/abstract.cfm?uri=oe-24-12-13091.

[3] D. Zhu, M. Guizar-Sicairos, B. Wu, A. Scherz, Y. Acremann, T. Tyliszczak, P. Fischer, N. Friedenberger, K. Ollefs, M. Farle, et al., Phys. Rev. Lett. **105**, 043901 (2010), URL https://link.aps.org/doi/10.1103/PhysRevLett.105.043901.

[4] H.-K. Lyeo and D. G. Cahill, Phys. Rev. B **73**, 144301 (2006), URL https://link.aps.org/doi/10.1103/PhysRevB.73.144301.

[5] R. M. Costescu, M. A. Wall, and D. G. Cahill, Phys. Rev. B **67**, 054302 (2003), URL https://link.aps.org/doi/10.1103/PhysRevB.67.054302.

[6] B. C. Gundrum, D. G. Cahill, and R. S. Averback, Phys. Rev. B **72**, 245426 (2005), URL https://link.aps.org/doi/10.1103/PhysRevB.72.245426.

[7] B. L. Henke, E. M. Gullikson, and J. C. Davis, Atomic Data and Nuclear Data Tables **54**, 181 (1993), ISSN 0092-640X, URL https://www.sciencedirect.com/science/article/pii/S0092640X83710132.

[8] M. A. Ordal, R. J. Bell, R. W. Alexander, L. A. Newquist, and M. R. Querry, Appl. Opt. **27**, 1203 (1988), URL http://www.osapublishing.org/ao/abstract.cfm?URI=ao-27-6-1203.

[9] K. M. McPeak, S. V. Jayanti, S. J. P. Kress, S. Meyer, S. Iotti, A. Rossinelli, and D. J. Norris, ACS Photonics **2**, 326 (2015), URL https://doi.org/10.1021/ph5004237.

[10] A. D. Avery, S. J. Mason, D. Bassett, D. Wesenberg, and B. L. Zink, Phys. Rev. B **92**, 214410 (2015), URL https://link.aps.org/doi/10.1103/PhysRevB.92.214410.

[11] M. A. Ordal, R. J. Bell, R. W. Alexander, L. L. Long, and M. R. Querry, Appl. Opt. **24**, 4493 (1985), URL http://www.osapublishing.org/ao/abstract.cfm?URI=ao-24-24-4493.

[12] M. A. Ordal, R. J. Bell, R. W. Alexander, L. L. Long, and M. R. Querry, Appl. Opt. **26**, 744 (1987), URL http://www.osapublishing.org/ao/abstract.cfm?URI=ao-26-4-744.

[13] P. B. Johnson and R. W. Christy, Phys. Rev. B **9**, 5056 (1974), URL https://link.aps.org/doi/10.1103/PhysRevB.9.5056.

[14] S. Srichandan, phd (2018).

[15] S. F.-Y. ZHU Li-Dan and S. F.-Y. ZHU Li-Dan, Chinese Physics Letters **29**, 66301 (2012), ISSN 0256-307X, URL http://cpl.iphy.ac.cn/EN/10.1088/0256-307X/29/6/066301.

[16] C. Schinke, P. Christian Peest, J. Schmidt, R. Brendel, K. Bothe, M. R. Vogt, I. Kröger, S. Winter, A. Schirmacher, S. Lim, et al., AIP Advances **5**, 067168 (2015), https://doi.org/10.1063/1.4923379, URL https://doi.org/10.1063/1.4923379.

[17] A. McConnell, S. Uma, and K. Goodson, Journal of Microelectromechanical Systems **10**, 360 (2001).

[18] J. Kimling, A. Philippi-Kobs, J. Jacobsohn, H. P. Oepen, and D. G. Cahill, Phys. Rev. B **95**, 184305 (2017), URL https://link.aps.org/doi/10.1103/PhysRevB.95.184305.

[19] G. Chen and P. Hui, Applied Physics Letters **74**, 2942 (1999), https://doi.org/10.1063/1.123973, URL https://doi.org/10.1063/1.123973.
